# Supplementary figures and images for: Identification and Typing of Strains of Wood-Rotting Basidiomycetes by Protein Profiling Using MALDI-TOF MS
Source: BioTech (Basel). 2022 Jul 27;11(3):30. doi: 10.3390/biotech11030030 (PMC9397059; doi:10.3390/biotech11030030)

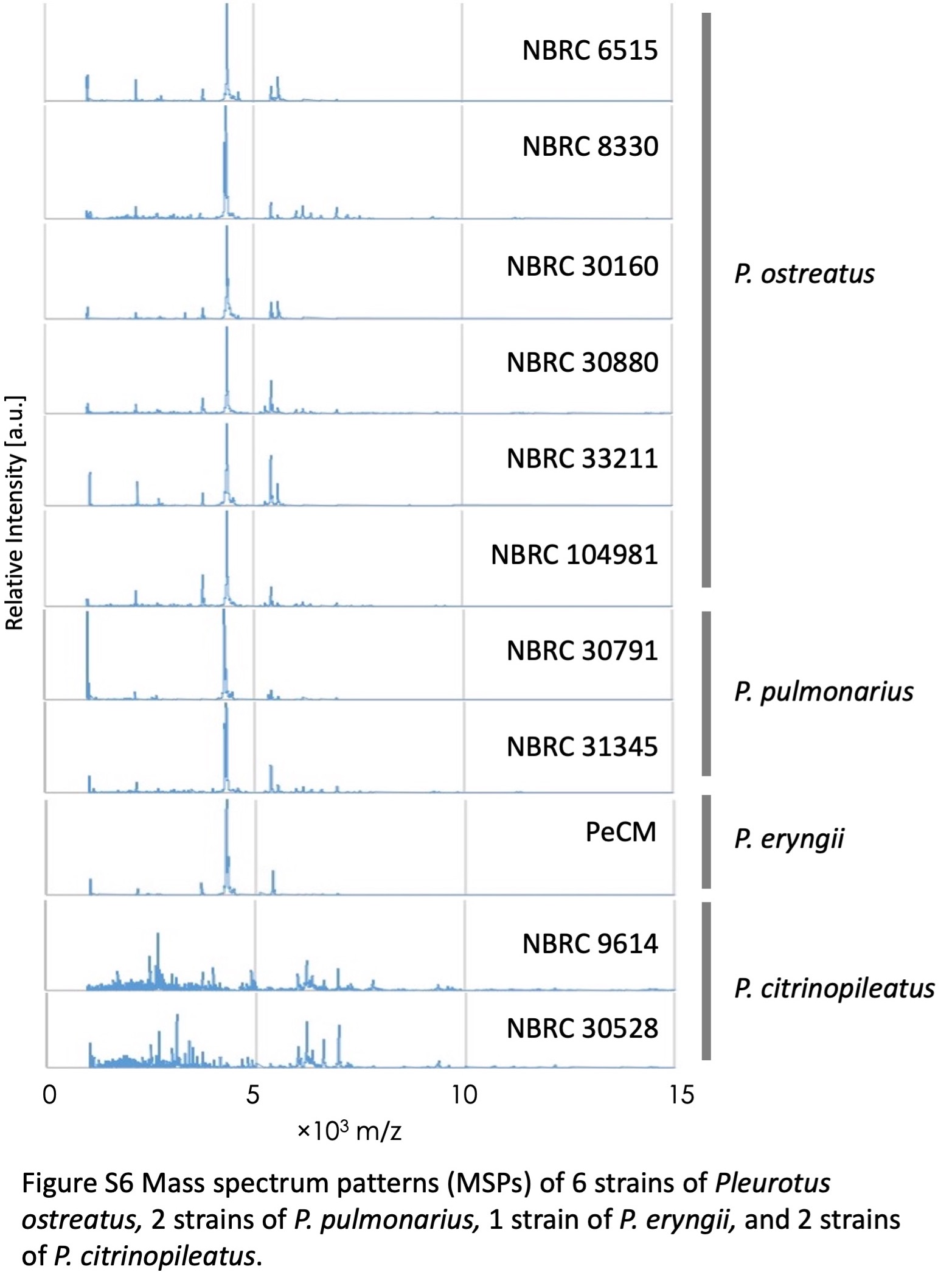

Supplement: Supplementary file 1 [file biotech-11-00030-s001.zip › Fig S6.jpg]

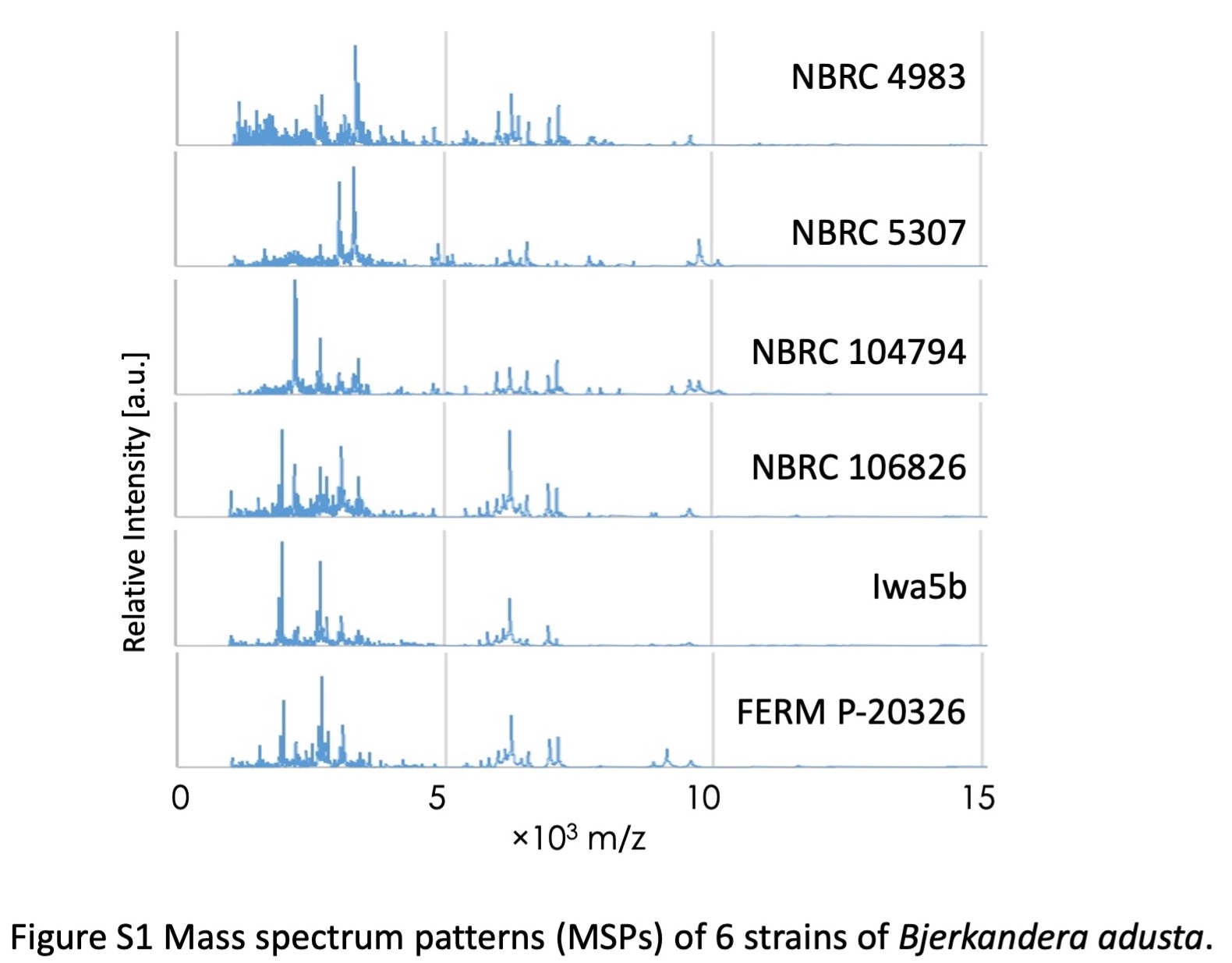

Supplement: Supplementary file 1 [file biotech-11-00030-s001.zip › Fig S1.jpg]

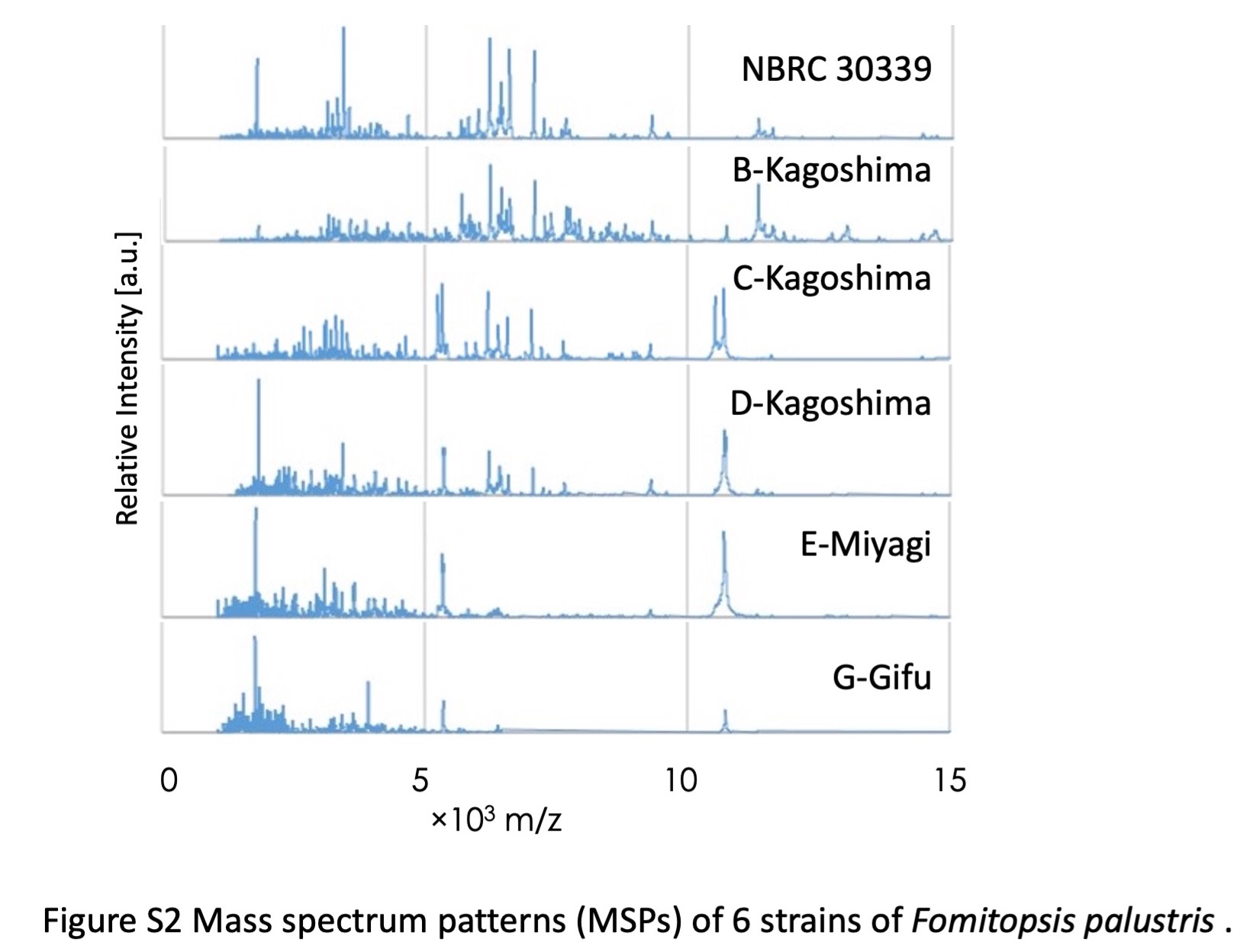

Supplement: Supplementary file 1 [file biotech-11-00030-s001.zip › Fig S2.jpg]

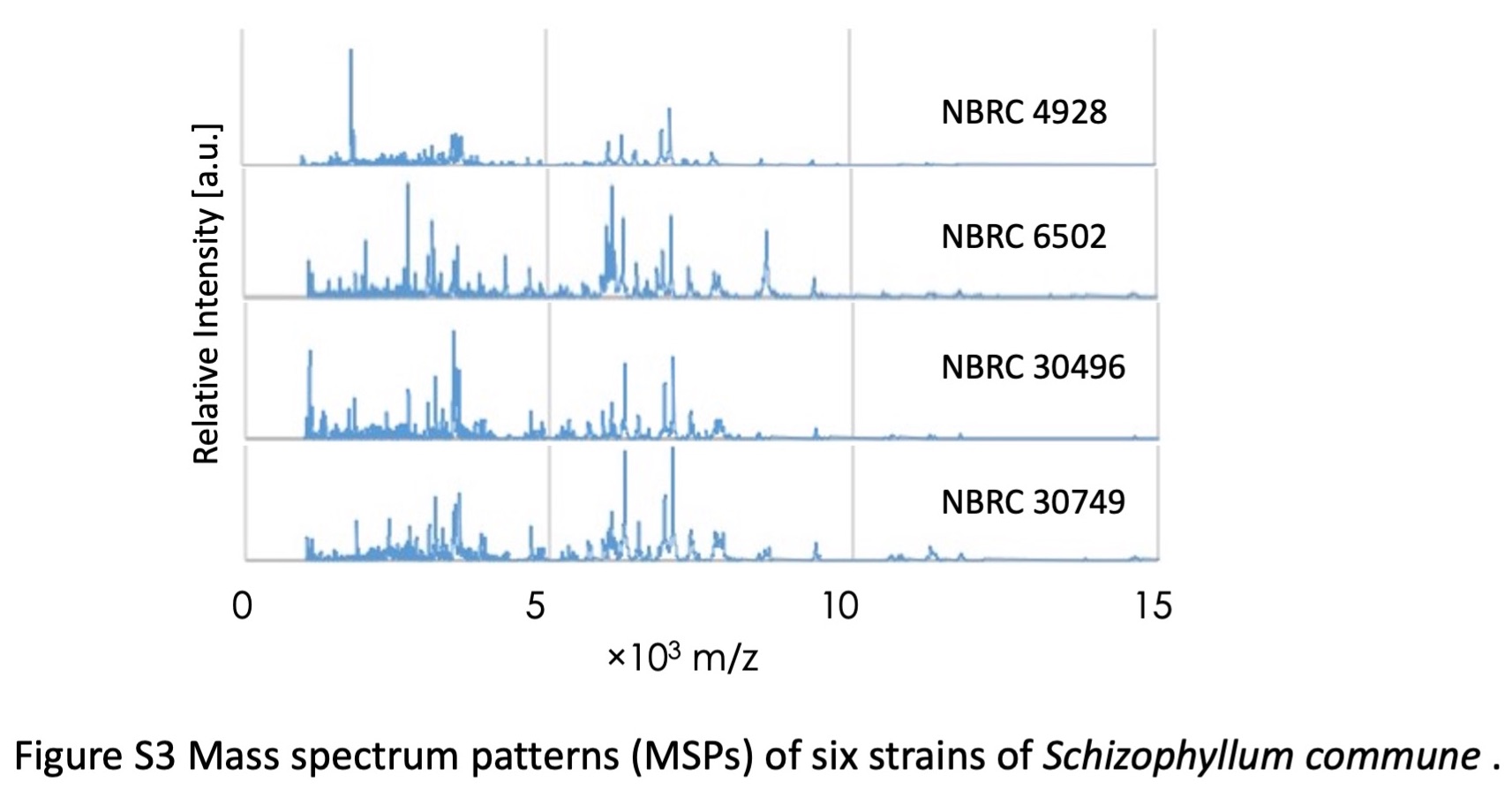

Supplement: Supplementary file 1 [file biotech-11-00030-s001.zip › Fig S3.jpg]

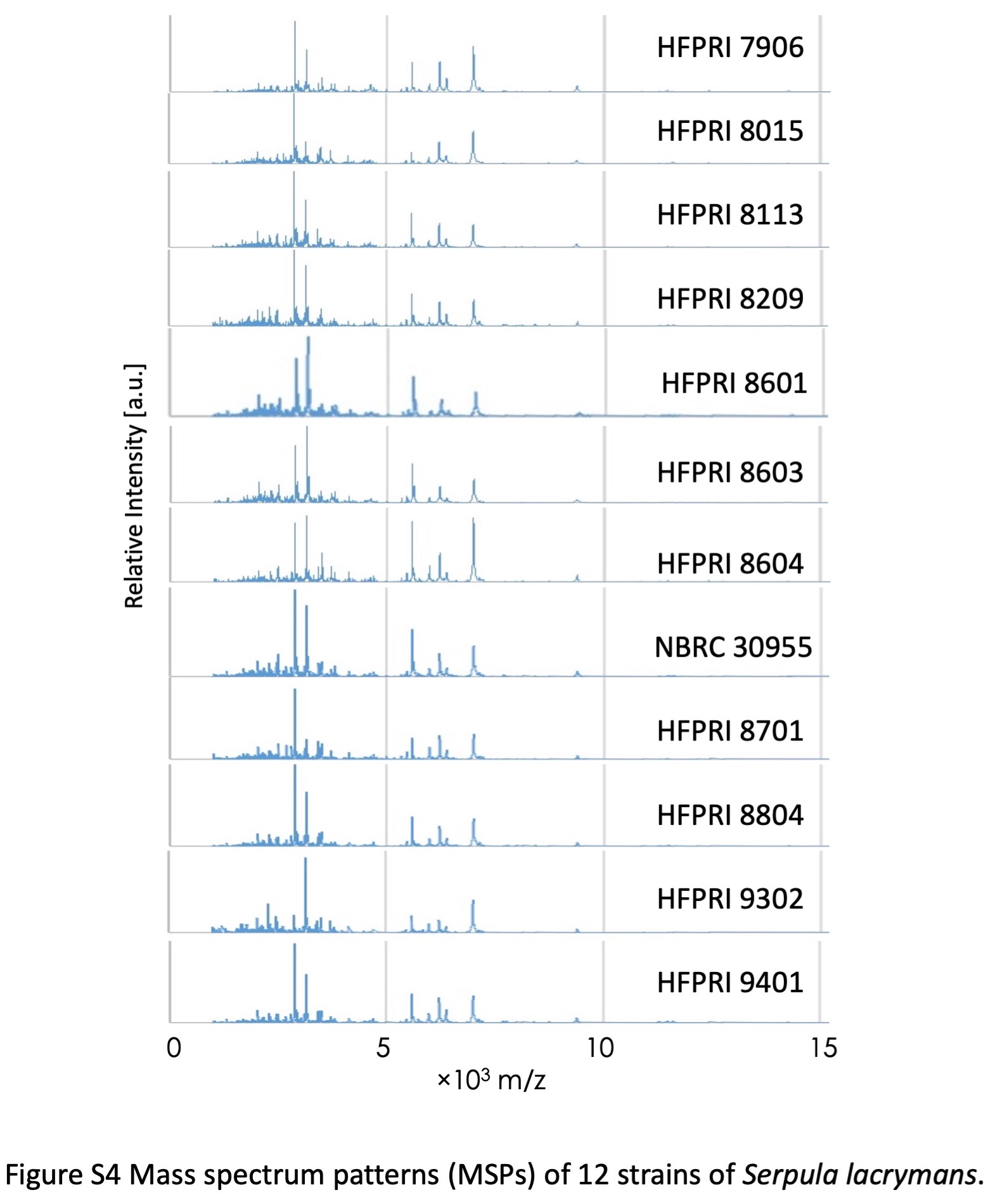

Supplement: Supplementary file 1 [file biotech-11-00030-s001.zip › Fig S4.jpg]

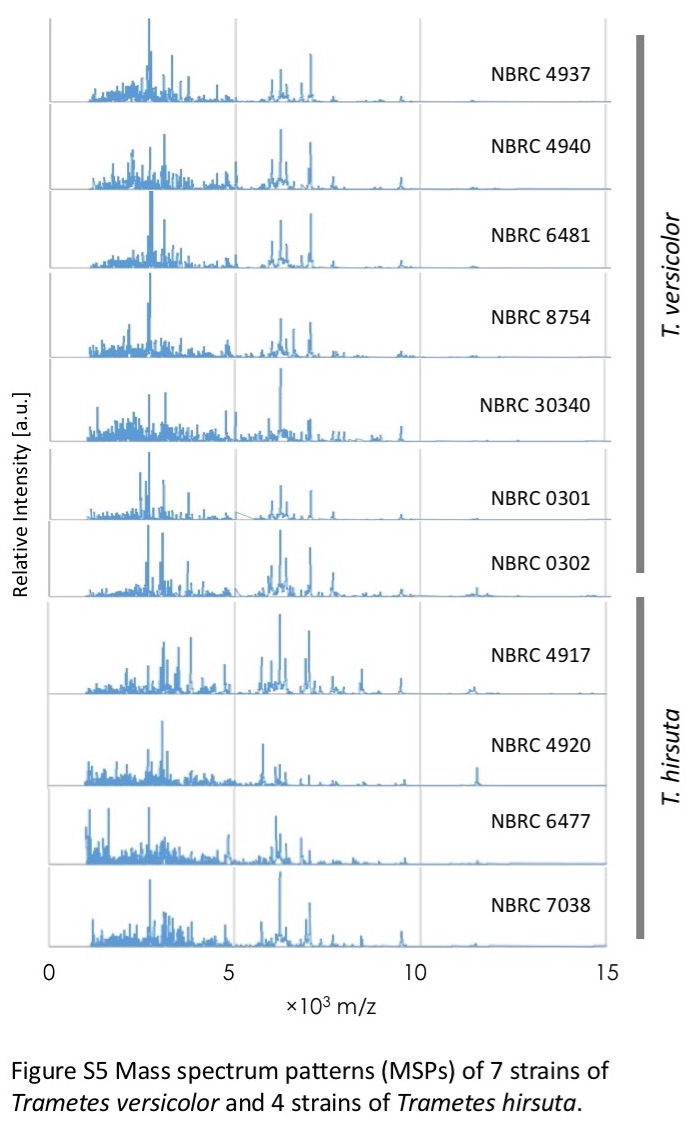

Supplement: Supplementary file 1 [file biotech-11-00030-s001.zip › Fig S5.jpg]
